# Inhibition of differentiation of monocyte-derived macrophages toward an M2-Like phenotype May Be a neglected mechanism of β-AR receptor blocker therapy for atherosclerosis

**DOI:** 10.3389/fphar.2024.1378787

**Published:** 2024-06-05

**Authors:** Shouyi Liu, Bo Zhang, Jingqun Zhou, Jianfeng Lv, Jinxia Zhang, Xiangyan Li, Weihua Yang, Yuanlin Guo

**Affiliations:** Affiliated Renhe Hospital, China Three Gorges University, Yichang, China

**Keywords:** β-AR blockers, atherosclerosis, macrophage polarization, inflammation, treatment 1.Introduction

## Abstract

The clinical efficacy of adrenergic β-receptor (β-AR) blockers in significantly stabilizing atherosclerotic plaques has been extensively supported by evidence-based medical research; however, the underlying mechanism remains unclear. Recent findings have highlighted the impact of lipid-induced aberrant polarization of macrophages during normal inflammatory-repair and regenerative processes on atherosclerosis formation and progression. In this review, we explore the relationship between macrophage polarization and atherosclerosis, as well as the influence of β-AR blockers on macrophage polarization. Based on the robust evidence supporting the use of β-AR blockers for treating atherosclerosis, we propose that their main mechanism involves inhibiting monocyte-derived macrophage differentiation towards an M2-like phenotype.

## 1 Introduction

Atherosclerosis is a chronic inflammatory disease occurring in the intima of large and medium-sized arteries. Many studies have shown that atherosclerosis is associated with oxidized low-density lipoprotein (ox-LDL), mainly driven by myeloid monocytes and macrophages to form an immune response (Flynn et al., 2019; Libby and Hansson, 2019; Wolf and Ley, 2019), However, the precise mechanism underlying atherosclerosis remains unclear. Clinically, atherosclerotic plaques can rupture due to an intense inflammatory response within the plaque, triggering activation of the coagulation system and localized thrombus formation, ultimately leading to severe acute ischemic events in vital organs.

A substantial body of evidence supports the efficacy of adrenergic beta receptor (β-AR) blockers in significantly stabilizing atherosclerotic plaques; however, the underlying mechanism remains poorly understood, and there is a dearth of scientific explanation for the necessity of early, long-term, and adequate administration of β-AR blockade to achieve plaque stabilization. Several studies have postulated potential factors contributing to the efficacy of β-AR blockers (Wikstrand et al., 2003; Vrablik et al., 2022): 1) The lipid solubility of certain β-AR blockers, leading to β-AR blockade in the central nervous system and decreased peripheral sympathetic nerve activity; 2). β1-AR blockade in the heart, reducing heart rate, blood pressure, and contractility, leading to beneficial hemodynamic changes; 3). The blockade of β-AR in biochemical systems confers endothelial protection, enhances nitric oxide and prostacyclin production, inhibits platelet aggregation, reduces the affinity of LDL for vascular wall proteoglycan, and suppresses smooth muscle cell proliferation. However, considerable controversy persists.

Recently, numerous studies have revealed the involvement of β2-AR in various inflammatory response processes within the body (Itoh et al., 2004; Tan et al., 2007; Kizaki et al., 2008a). The driver cells of atherosclerotic inflammation are mainly macrophages, which are rich in β2-AR on their surface, and β2-AR is the main functional β-AR in macrophages (Lamkin et al., 2016). It is highly likely that β-AR blockers affect the inflammatory process of atherosclerotic plaques by regulating the function of macrophages in atherosclerosis plaques through β2-AR. Our research group reviewed the relationship between macrophage polarization and atherosclerosis formation and the related research literature on the effect of β-AR blockers on macrophage polarization, as well as the evidence-based medical evidence of β-AR blockers in the treatment of atherosclerosis. We suggest that inhibition of M2-like differentiation of monocyte-derived macrophages by β-AR blockers may be the main mechanism by which β-AR blockers treat atherosclerosis.

## 2 Lipids disrupt the physiological process of monocyte-derived macrophage polarization program, impeding normal inflammatory-repair and regenerative mechanisms and ultimately contributing to the development of atherosclerosis

### 2.1 Macrophage polarization is a prevalent phenomenon observed in macrophages during the inflammation-repair and regeneration processes triggered by various injuries (Parisi et al., 2018). Throughout the course of inflammation-repair and regeneration, monocytes undergo differentiation into macrophages, and adherence to the established macrophage polarization program is an essential prerequisite for successful tissue repair (Wynn and Vannella, 2016; Funes et al., 2018; Oishi and Manabe, 2018; Parisi et al., 2018)

**FIGURE 1:**
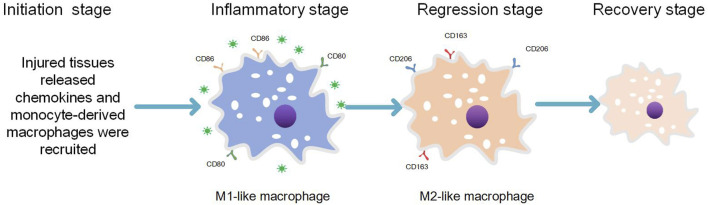
The normal inflammation-repair and regeneration process includes four stages.

In addition to their role in host defense, monocyte-derived macrophages also play a crucial role in tissue development, maintenance of tissue homeostasis, and tissue regeneration (Oishi and Manabe, 2018). During the process of normal and complete repair of damaged tissues following ordinary injury, four stages are involved: initiation, inflammation, abatement, and integrity restoration (Funes et al., 2018) (see Figure 1). 1) Initiation phase: Circulating monocytes migrate into the injured tissue under the chemotactic effect of inflammatory factors (Blériot et al., 2020) released by resident macrophages present as the first line of defense. 2) Inflammation phase: Monocytes at the site of tissue injury undergo differentiation into pro-inflammatory phenotypes expressing M1 markers. This leads to elimination of causative factors through inflammatory responses generated by pro-inflammatory macrophages and damage-associated molecular patterns (DAMPs) released by necrotic cells within the tissue. 3) Inflammation attenuation phase: Once pathogenic factors and DAMPs are completely cleared, recruitment of monocytes to the lesion ceases. Simultaneously, already recruited pro-inflammatory macrophages differentiate into anti-inflammatory macrophages expressing M2 markers. 4) Integrity restoration phase: Monocyte-derived M2-like phenotypic macrophages along with other tissue-resident macrophages expressing M2 markers contribute to completing the repair process (Wynn and Vannella, 2016; Funes et al., 2018; Oishi and Manabe, 2018; Parisi et al., 2018). Macrophages in the inflammation-repair and regeneration process exhibit either pro-inflammatory (expressing M1 markers) or anti-inflammatory phenotypes (expressing M2 markers) as a result of macrophage polarization, which is driven by changes in the pathologic microenvironment that align with the demands of the inflammation-repair and regeneration response phase (Parisi et al., 2018). Macrophage polarization represents an inherent property of macrophages involved in various injury-induced inflammation-repair and regeneration processes (Parisi et al., 2018). Disturbance of the normal macrophage polarization program leads to sustained recruitment of pro-inflammatory macrophages with an M1-like phenotype, exacerbating tissue injury.

Recently, the application of single-cell sequencing technology has provided a better characterization of macrophage heterogeneity. A comprehensive analysis of data obtained from this technology in the study of atherosclerosis reveals that macrophages in endothelial atherosclerotic plaques can now be more clearly classified into two main groups: pro-inflammatory types expressing M1 markers and anti-inflammatory types expressing M2 markers (Willemsen and de Winther, 2020; Farahi et al., 2021). While some researchers have suggested abandoning the classification of macrophages into M1-like phenotype or M2-like phenotype (Nahrendorf and Swirski, 2016). It cannot be denied that macrophage polarization plays an objective role in the inflammation-repair and regeneration process. Atherosclerosis itself represents a specific inflammatory-repair and regenerative response process, and therefore, the implications of the M1-like phenotype and M2-like phenotype classification paradigm are still relevant for comparing differences between macrophage polarization programs during normal inflammatory-repair and regenerative processes and after monocyte-to-macrophage differentiation in the course of atherosclerosis to explore mechanisms underlying its development (Martinez and Gordon, 2014; Libby and Hansson, 2019; Libby, 2021; Mushenkova et al., 2022). Currently, there is no official dissenting opinion on categorizing macrophages as M1/M2 phenotypes within the context of inflammatory-repair responses (Martinez and Gordon, 2014; Libby, 2021).

### 2.2 Lipid-induced macrophage differentiation towards M2-like phenotype interferes with the normal polarization program of macrophages during inflammation-repair and regeneration and promotes atherosclerosis formation

Cytological studies have revealed that lipids can induce macrophage differentiation towards an M2-like phenotype (Sokolov et al., 2014; Seo et al., 2015; Pireaux et al., 2016; Lin et al., 2021; Zhang et al., 2023). Previous investigations have demonstrated that approximately 22% of subendothelial macrophages exhibit characteristics of “M2” or “selectively activated” macrophages after phagocytosis of lipids (Taleb, 2016). Pathological examinations have also confirmed the predominance of an M2-like phenotype in early atherosclerotic plaques (containing fatty streaks) (Khallou-Laschet et al., 2010; Moore et al., 2013). Monocytes play a crucial role as an early source of foam cells (Randolph, 2014; Guilliams et al., 2018). The shoulder region of atherosclerotic plaques exhibited a significant presence of pro-inflammatory macrophages expressing M1 markers (Stöger et al., 2012), with particular prominence observed in the shoulder area of unstable atherosclerotic plaques. Recently conducted macrophage kinetic studies have confirmed that newly recruited monocyte-derived macrophages stay at the plaque edge and do not penetrate significantly deeper into the plaque. Newly recruited macrophages, together with macrophages recruited to the plaque Previously, showed a hierarchical distribution like the annual rings of a tree. Within this distribution, foamy macrophages can be observed among the previously recruited ones located in the plaque area (Williams et al., 2018). Therefore, cytological investigations have revealed that lipids can induce differentiation of macrophages towards an M2-like phenotype both *in vitro* and *in vivo*. Modern research techniques have further validated that pro-inflammatory macrophages as well as lipid-containing foamy macrophages exhibit a regional distribution within atherosclerotic plaques, including both lipid-streaked plaques and typical atherosclerotic plaques. The formation of foam cells by pro-inflammatory macrophages upon contact with lipids serves as an indicator for initiating atherogenesis, suggesting that lipid-induced differentiation of monocyte-derived macrophages to an M2-like phenotype plays a role in the development of atherosclerosis.

When lipids accumulate beneath the intima, lipid-induced cytotoxicity leads to tissue damage, thereby triggering the activation of resident macrophages within the tissue. These activated macrophages subsequently release inflammatory chemokines to initiate an inflammatory response (Blériot et al., 2020). Monocytes differentiate into macrophages and are recruited to the site of tissue injury, marking the onset of phase 2 in the inflammation-repair and regeneration process known as the inflammatory response phase. Initially expected to exhibit pro-inflammatory characteristics with M1 markers and contribute to tissue damage, monocyte-derived macrophages undergo transformation into inflammation-suppressive macrophages expressing M2 markers upon exposure to lipids. Consequently, this phenomenon of cell transformation occurs earlier than anticipated during the inflammatory response phase under lipid influence, resulting in lipid processing by M2-like phenotypic macrophages that lack direct capacity for generating an inflammatory response. Thus, contact with lipids leads monocyte-derived macrophages to transform into anti-inflammatory macrophages expressing M2 markers. As a result, under lipid influence, cell transformation takes place prematurely during the inflammatory stage instead of after completion of phase 2; thereby enabling lipid processing without direct induction of an inflammatory response.

**FIGURE 2:**
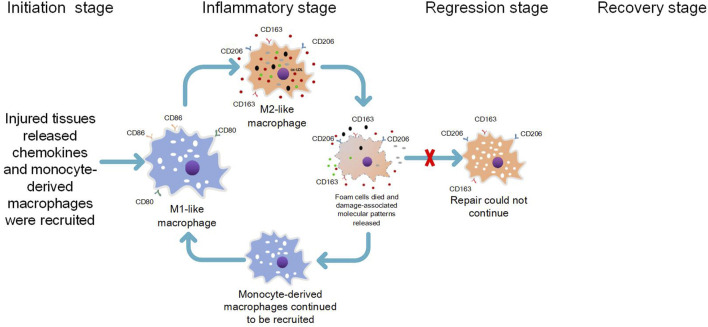
The pathological process of the formation and progression of atherosclerosis.

M2-like phenotype macrophages exhibit a robust phagocytic capacity (Radhika and Sudhakaran, 2013), yet they possess a diminished ability to process lipids compared to M1-like phenotype macrophages (Viola and Soehnlein, 2015). Following lipid phagocytosis, M2-like phenotype macrophages are easily induced by lipids to transform into anti-inflammatory foam macrophages with an M2-like phenotype (van et al., 2011; Oh et al., 2012; Kim et al., 2018), Unlike pro-inflammatory macrophages that engulf common pathogenic agents or DAMPs (Cochain and Zernecke, 2017), foam macrophages do not immediately trigger a strong inflammatory response. Consequently, early atherosclerotic lipid-rich plaques generally remain stable (Khallou-Laschet et al., 2010). However, the accumulation of foamy macrophages is positively associated with the severity of atherosclerosis (Kim et al., 2018), posing as an underlying risk for further lesion development. It has been observed that lipid phagocytosis by M2-like phenotype macrophages can elicit a pro-inflammatory response (van et al., 2011). Moreover, these cells display lower tolerance towards lipids than M1 macrophages and are prone to die (Isa et al., 2011). Our research group has proposed that this phenomenon may be attributed to the persistent phagocytosis of lipids by M2-like phenotype foam cells, which can activate the formation of NLRP3 inflammatory vesicles. This activation initiates the pyroptosis pathway and leads to the release of IL-18 and IL-1β from dying foam cells (Lin et al., 2013; Zhang et al., 2023). The chemotactic effect of IL-1β on monocyte-derived macrophages is well-known in promoting atherosclerosis formation in coronary arteries, as its levels have been shown to correlate with disease severity (Rajamäki et al., 2010; Mai and Liao, 2020). Additionally, besides apoptosis, focal death associated with cell membrane rupture can occur in foam cells due to activation of specific death pathways such as pyroptosis. This process further promotes atherosclerosis progression (Lin et al., 2013; Zhang et al., 2023). Furthermore, when foam cells undergo pyroptotic death, they trigger a pro-inflammatory response and contribute to atherosclerosis progression through cell membrane rupture and release of DAMPs. These DAMPs are then phagocytosed by inflammation-suppressing property M2-like phenotypic macrophages and accumulate within them (Cochain and Zernecke, 2017; Zhang et al., 2023). Considering that lipids persist within plaques without being removed, our research group concluded that a vicious cycle occurs during atherosclerosis progression involving recruitment and differentiation of M1-type macrophages into M2-like phenotypes followed by phagocytosis and subsequent death processes. This cycle perpetuates itself by recruiting more M1-type macrophages for differentiation into M2-like phenotypes followed by phagocytosis and death (Zhang et al., 2023) (see Figure 2). Excessive release of inflammatory factors from foam cells within the atherosclerotic plaque, accompanied by cell membrane rupture-associated death, leads to recruitment of M1-like phenotype pro-inflammatory macrophages to the shoulder region of the plaque due to potent chemotaxis. The ensuing inflammatory response initiated by these M1-like phenotype pro-inflammatory macrophages may result in plaque rupture at the shoulder, activation of the coagulation system, and localized thrombus formation, ultimately leading to severe acute ischemic events in vital organs.

1) The lipid appears in the subintima, and the lipid toxicity causes tissue damage, which first triggers the tissue-resident macrophages to release inflammatory chemokines and initiate the recruitment response; 2) Circulating monocytes enter the tissue by chemotactic action of inflammatory factors. Monocyte-derived macrophages are supposed to process lipid and injured tissue by expressing the proinflammatory functional characteristics of the M1-like phenotype. However, monocyte-derived macrophages are transformed into anti-inflammatory macrophages expressing M2-like phenotype due to their exposure to lipids. 3) After phagocytosis of lipids, M2-like phenotypic foam macrophages with anti-inflammatory properties are formed, and atherosclerosis is formed. M2-like phenotypic foam macrophages act like tissue-resident macrophages that are first damaged by lipids, releasing inflammatory chemokines and even death associated with cell membrane rupture (e.g., pyroptosis), releasing damage-associated molecular patterns (DAMPs) that continue to initiate recruitment responses. 4) Monocyte-derived macrophages continue to be recruited and are supposed to treat lipids and damaged tissues with the pro-inflammatory functional characteristics of expressing M1 markers, but they are again converted by lipids into anti-inflammatory macrophages expressing M2 markers to continue the above pathological cellular process.

## 3 Inhibition of aberrant polarization of monocyte-derived macrophages during the inflammatory-repair and regenerative process in atherosclerosis and its potential for preventing atherosclerosis progression

Currently, the conventional perspective is based on cell physiology, which suggests that M1-like phenotypic macrophages primarily exhibit pro-inflammatory properties and can induce tissue damage, while anti-inflammatory M2-like phenotypic macrophages can suppress tissue inflammation and facilitate tissue repair. Consequently, it is inferred that promoting differentiation towards M2-like macrophages could inhibit atherosclerosis development (Mantovani et al., 2009; Khallou-Laschet et al., 2010; Colin et al., 2014; Viola and Soehnlein, 2015). In studies involving mouse models of atherosclerosis regression (including mouse transplantation models and reverse mouse models), it was observed that the regression of atherosclerosis coincided with a significant increase in the population of inflammation-suppressive M2-like phenotypic macrophages. These findings were considered supportive of the traditional viewpoint (Chistiakov et al., 2017). However, earlier research has demonstrated that pioglitazone promotes further necrotic core development within aortic root-formed atherosclerotic plaques in high-fat diet-induced Ldlr−/− mice by promoting macrophage differentiation towards an M2-like phenotype (Thorp et al., 2007) and enhancing polarization towards an M2-like phenotype (Mamilos et al., 2023), In contrast to this observation, our group discovered (Zhang et al., 2023) that in more severe patches of atherosclerotic plaques, although there was an increased proportion of macrophages exhibiting an M1-like phenotype constitutively present; there was also a similar absolute increase in the number of macrophages displaying an M2-like phenotype. This finding does not reflect any inhibitory effect on the progression of atherosclerosis. Revised sentence: Monocyte-derived macrophages phagocytose lipids, undergo aberrant polarization, and form foam cells, similar to tissue-resident macrophages that release various pro-inflammatory mediators upon lipid exposure. In severe cases, foam cells undergo direct programmed death while recruited monocyte-derived macrophages cause tissue damage through the release of injurious inflammatory factors (Blériot et al., 2020); Therefore, it is crucial to inhibit the inflammatory response of monocyte-derived macrophages during this period in order to prevent their differentiation towards an M2-like phenotype. It has been observed that excessive cholesterol uptake by macrophages induces endoplasmic reticulum stress and promotes their differentiation towards an M2-like phenotype, thereby facilitating atherosclerosis progression (Oh et al., 2012), Additionally, inhibiting the differentiation of monocyte-derived macrophages towards an M2-like phenotype may impede the progression of atherosclerosis. Our research group posits that the regression of the aforementioned mouse model of atherosclerosis is attributed to the artificial removal of hyperlipidemic conditions and subsequent elimination of pathogenic factors. By eliminating the hyperlipidemic environment during the inflammatory response phase in lipid inflammation-repair and regeneration, monocyte-derived macrophages are not induced to differentiate into an anti-inflammatory M2-like phenotype, thereby obviating any potential interference with normal macrophage polarization programming by lipids. (The observation of a substantial number of anti-inflammatory M2-like macrophages in the regression model of atherosclerosis in mice occurs subsequent to their departure from the hyperlipidemic environment), which serves as the underlying cause for atherosclerosis regression.

## 4 Inhibition of lipid-induced M2-like differentiation of monocyte-derived macrophages to inhibit atherosclerosis progression by β-AR receptor blocking agents

### 4.1 Exploration of evidence-based medical evidence mechanisms for stabilization of AS plaques by β-AR blockers

Over the past 4 decades, data from numerous experimental animal studies have consistently indicated that β-AR blockers possess anti-atherosclerotic effects (Pick and Glick, 1977; Spence et al., 1984; Wikstrand et al., 2003; Ulleryd et al., 2014; Chen et al., 2019). In both primary and secondary prevention of coronary heart disease, β-blockers have demonstrated superior efficacy in terms of their anti-atherosclerotic effects according to multiple clinical studies (Sipahi et al., 2007; Vrablik et al., 2022). Furthermore, several other investigations have reported various benefits associated with β-AR blockers in patients with coronary artery disease through their anti-atherosclerosis properties (Ellison and Gandhi, 2005). Initially, it was believed that the attenuation of myocardial contractility and reduction in heart rate resulting from β1 receptor blockade were crucial mechanisms for preventing plaque rupture by decreasing blood flow shear stress and minimizing damage to the vascular endothelium (Kaplan and Manuck, 1994), However, subsequent findings revealed that even after adjusting for mean heart rate during treatment, the association between β-AR blockers and a reduced rate of atherosclerosis progression remained statistically significant. Several studies have suggested that heart rate reduction is not the sole mechanism responsible for the beneficial effects of β-AR blockers on atherosclerosis (Kaplan and Manuck, 1994; Sipahi et al., 2007). Experimental animal studies have demonstrated that Niprodilol, a non-selective β-blocker with nitroglycerin-like activity, exerts a significant anti-atherosclerotic effect independent of its lipid-lowering and blood pressure-reducing properties by reducing inflammatory cells (such as macrophages and T-lymphocytes) within atherosclerotic plaques in rabbits. In contrast, atenolol, a highly selective β1-adrenergic receptor blocker, along with nitric oxide donors (nitroglycerin and isosorbide nitrate), did not exhibit similar effects. These findings strongly suggest that the anti-atherosclerosis mechanism of nipradilol is unrelated to β1-adrenergic receptor blockade or the promotion of nitric oxide release (Thakur et al., 2002). Clinical studies have also reported an increased risk of stroke in elderly patients treated with atenolol (Vrablik et al., 2022).

As the understanding of the crucial role of reducing inflammation in atherosclerotic plaques for stabilizing them has gradually emerged, β-AR blockers have gained increasing recognition for their potential anti-atherosclerotic effects through direct beneficial actions on the arterial wall (Kizaki et al., 2008b). Simultaneously, evidence-based medicine has also revealed that not all β-AR antagonists exhibit analogous beneficial properties (Ellison and Gandhi, 2005). Currently, propranolol, metoprolol, and carvedilol are the only β-AR antagonists supported by clear evidence-based medicine. These agents are not highly selective β1-AR antagonists but instead display varying degrees of selectivity towards β2-AR (Smith and Teitler, 1999; Hoffmann et al., 2004; Baker, 2005; Chen et al., 2019), suggesting that the mechanism underlying their ability to reduce inflammation in atherosclerotic plaques may involve blocking of β2-adrenergic receptors. Genetic studies investigating the association between polymorphisms in the β2-adrenergic receptor gene and acute coronary syndromes (ACS) have concluded that there is a close correlation between β2-adrenergic receptors and ACS development (Lanfear et al., 2005; Jaillon and Simon, 2007).

### 4.2 β-AR receptor blockers, via β2-AR, inhibit lipid-induced macrophage differentiation from M1-like phenotype to M2-like phenotype and inhibit atherosclerosis progression (Zhang et al., 2023)

Our previous study demonstrated that β2-AR stimulators can enhance the effects of ox-LDL on macrophage function. However, metoprolol, propranolol (Smith and Teitler, 1999; Hoffmann et al., 2004; Baker, 2005) and ICI118551, which exhibit varying degrees of selectivity for β2-AR, can transduce signals through β2-AR and inhibit the effects of ox-LDL on macrophage function. Notably, highly selective β2-AR blocker ICI118551 exerts the most potent effect (Guo et al., 2014). Interestingly, our findings suggest that these aforementioned β2-AR blockers exert immunomodulatory effects on macrophages via a mechanism beyond pure blockade but rather act as inverse agonists relative to β2-AR stimulators (Guo et al., 2014), Similar observations have been reported in other studies (Grisanti et al., 2019).

It has long been established that β2-AR stimulants, acting through the β2-AR receptor, promote macrophage differentiation towards an M2-like phenotype while inhibiting macrophage differentiation towards an M1-like phenotype. Conversely, β-AR blockers, via the β2-AR receptor, induce macrophage differentiation towards an M1-like phenotype and inhibit differentiation towards an M2-like phenotype (Itoh et al., 2004). In our study group, Apoe−/− mice were fed a high-fat diet (Zhang et al., 2023) and subsequently treated with either the β2-AR stimulant salbutamol (SAL) or the β2-AR blocker ICI118551 (ICI). The number of M2-like phenotype macrophages was used as the independent variable in our methodology analysis with atherosclerotic plaque formation serving as the dependent variable. We identified a causal relationship between early inhibition of macrophage differentiation into an M2-like phenotype and suppression of atherosclerosis development. Atherosclerotic plaque formation was successfully induced in four experimental groups: control group on normal diet; model group on high-fat diet; SAL-treated group on high-fat diet; and ICI-treated group on high-fat diet. However, the severity of atherosclerotic plaque formation in each group was the most severe in SAL group, followed by model group, ICI group and experimental control group. The model and SAL groups exhibited significant formation of atherosclerotic plaques in the aortic root, whereas no notable atherosclerosis plaques were observed in the ICI group and experimental control group. SAL prominently augmented ox-LDL levels to promote macrophage differentiation towards an M2-like phenotype, thereby facilitating atherosclerosis formation. Conversely, ICI, acting as an inverse agonist of SAL, inhibited the development of atherosclerosis. To elucidate the mechanism underlying this inhibition by ICI, an atherosclerotic cell model was employed. It was discovered that ICI exerted inhibitory effects on lipid-induced differentiation of M1-type macrophages into the M2-like phenotype and also suppressed activation of the pyroptosis pathway in macrophage-derived foam cells with an M2-like phenotype. This resulted in reduced transcription of IL-18 and IL-1β, prevention of foam cell pyroptosis, decreased release of DAMPs (damage-associated molecular patterns), all favorably contributing to inhibition of atherosclerosis progression. Meanwhile, our group discovered that metoprolol and propranolol exhibited a similar effect to ICI but demonstrated a more pronounced drug-dose-dependent relationship than ICI (Guo et al., 2014; Zhang et al., 2023). Recently, other animal experimental studies have revealed that pretreatment of β-AR blockers (such as ICI, Carv, and Met High) in experimental animals can reduce the degree of inflammatory response of leukocytes in the injured area to pathogenic factors (Grisanti et al., 2019), indicating that pre-treatment with β-AR blocker via β2-AR on atherosclerotic plaques can decrease the degree of polarization of M2-like phenotype or the number of M2-like phenotypes within the plaque. This reduction can lower pro-inflammatory factors released from damaged lipid-induced M2-like phenotype macrophages while decreasing recruited macrophage numbers and reducing intensity of inflammatory response. Therefore, this mechanism is also one important pharmacological approach by which β-AR blockade stabilizes atherosclerotic plaques.

One mechanism of action for β-AR blockers with varying degrees of selectivity for β2-AR is the inhibition, via β2-AR, of monocyte-derived M1-type macrophage recruitment. These macrophages are abnormally differentiated by lipids into an M2-like phenotype, which compromises proper lipid processing (a disease-causing factor) by M1-like phenotypic macrophages during the inflammatory response phase and hinders phagocytosis of lipids by M2-like phenotypic macrophages. By appropriately handling lipids (pathogenic factors) during the inflammatory response period and preventing their uptake by M2-like phenotype macrophages, β-AR blockers reduce activation of the pyroptosis pathway and subsequent release of IL-18 and IL-1β from lipid-phagocytosing M2-like phenotype macrophages, thereby decreasing foam cell death involving membrane rupture (e.g., pyroptosis). Consequently, these findings provide objective prior validation supporting the anti-atherosclerotic effects exerted by β-AR blockers in atherosclerosis treatment.

The proposed mechanism by which β-AR blockers stabilize atherosclerotic plaque in this review also offers a scientific and rational explanation for the “early, long-term, and sufficient clinical application principle” advocated by evidence-based medicine for β-AR blockade in stabilizing atherosclerotic plaque: “Early” refers to the necessity of administering β-AR blockers during the inflammatory phase of the inflammation-repair and regeneration process to prevent lipid-induced differentiation of M1 macrophages into M2-like phenotypes. Given that atherosclerosis follows a chronic course characterized by a “vicious cycle” (Zhang et al., 2023), each vicious cycle entails an inflammatory response period, and “long-term” implies pre-administration of β-AR blockers before each such period to reduce polarization or quantity of M2-like phenotype macrophages within atherosclerotic plaques. This prevents lipid-induced damage on M2-like macrophages leading to increased release of pro-inflammatory factors and mitigates the intensity of the inflammatory response. Lastly, “sufficient dose” denotes evidence-based medical support for non-selective β-AR blockade, with drug concentration demonstrating a significant dose-dependent relationship. Only through adequate dosage can full blocking effects on β2-AR be achieved while preventing lipid-induced differentiation of M1 macrophages into M2-like phenotypes.

## 5 Conclusion and prospect

We clarified that β2-AR blockers maintain monocyte-derived macrophages processing lipids in an M1-like phenotype by inhibiting lipid-induced differentiation of recruited monocyte-derived macrophages with M1-like phenotype as the key to stabilize atherosclerotic plaques. The understanding of the pathophysiological mechanisms by which pathogenic factors cause chronicity of inflammation by inducing differentiation of monocyte-derived M1-like phenotype macrophages to M2-like phenotype during the inflammatory response phase of the inflammation-repair and regeneration process is of general guidance for exploring the mechanisms of other non-infectious chronic inflammatory diseases in the clinic. The development of drugs that inhibit lipid-induced differentiation of monocyte-derived M1-like phenotype macrophages to M2-like phenotype more potently, or the development of drugs that promote the degradation of lipotoxicity by M1-like phenotype macrophages, will be helpful to change the current situation of clinical prevention and treatment of atherosclerotic progression.
